# Ecological Shifts of Supragingival Microbiota in Association with Pregnancy

**DOI:** 10.3389/fcimb.2018.00024

**Published:** 2018-02-15

**Authors:** Wenzhen Lin, Wenxin Jiang, Xuchen Hu, Li Gao, Dongmei Ai, Hongfei Pan, Chenguang Niu, Keyong Yuan, Xuedong Zhou, Changen Xu, Zhengwei Huang

**Affiliations:** ^1^Department of Endodontics, Ninth People's Hospital, Shanghai Jiao Tong University School of Medicine, Shanghai, China; ^2^Shanghai Key Laboratory of Stomatology and Shanghai Research Institute of Stomatology, National Clinical Research Center of Stomatology, Shanghai, China; ^3^Department of Information and Computational Sciences, University of Science and Technology Beijing, Beijing, China; ^4^State Key Laboratory of Oral Diseases, West China Hospital of Stomatology, Sichuan University, Chengdu, China; ^5^Obstetrics Department, Obstetrics and Gynecology Hospital of Fudan University, Shanghai, China

**Keywords:** supragingival plaque, oral microbiota, bacterial diversity, pregnancy, female steroid hormones, 16S rDNA sequencing

## Abstract

Pregnancy is a physiological process with pronounced hormonal fluctuations in females, and relatively little is known regarding how pregnancy influences the ecological shifts of supragingival microbiota. In this study, supragingival plaques and salivary hormones were collected from 11 pregnant women during pregnancy (P1, ≤14 weeks; P2, 20–25 weeks; P3, 33–37 weeks) and the postpartum period (P4, 6 weeks after childbirth). Seven non-pregnant volunteers were sampled at the same time intervals. The microbial genetic repertoire was obtained by 16S rDNA sequencing. Our results indicated that the Shannon diversity in P3 was significantly higher than in the non-pregnant group. The principal coordinates analysis showed distinct clustering according to gestational status, and the partial least squares discriminant analysis identified 33 genera that may contribute to this difference. There were differentially distributed genera, among which *Neisseria, Porphyromonas*, and *Treponema* were over-represented in the pregnant group, while *Streptococcus* and *Veillonella* were more abundant in the non-pregnant group. In addition, 53 operational taxonomic units were observed to have positive correlations with sex hormones in a redundancy analysis, with *Prevotella* spp. and *Treponema* spp. being most abundant. The ecological events suggest that pregnancy has a role in shaping an at-risk-for-harm microbiota and provide a basis for etiological studies of pregnancy-associated oral dysbiosis.

## Introduction

The oral microbiota comprises more than 700 bacterial species, and co-evolves over the long term with the host beginning at birth (Aas et al., 2005). The microorganisms survive daily physical and chemical stimuli from food intake and oral hygiene measures, resulting in a finely tuned ecosystem (Zaura et al., 2014). However, this natural state is not always easy to maintain and can be disturbed by numerous factors, most of which are host-derived. Host susceptibility may act as a trigger for polymicrobial oral diseases, including dental caries, periodontal diseases, and oral cancers (Griffen et al., 2012; Yang et al., 2012; Guerrero-Preston et al., 2016). Hormonal imbalances, such as those observed with diabetes and obesity, can also contribute to the development of dysbiosis (Stabholz et al., 2010).

Pregnancy is a physiological perturbation with pronounced hormonal fluctuations in females. As a multifactorial disease, pregnancy gingivitis is still common and affects at least 35% of pregnant women (Hasson, 1960). It is well-acknowledged that the prevalence and severity of gingivitis are greater in pregnant women than in their non-pregnant counterparts (Gürsoy et al., 2008). The underlying mechanisms have been investigated since the 1960s (Loe and Silness, 1963). It is supposed that gingival inflammation is initiated by periodontal pathogens and exacerbated by endogenous steroid hormones during gestation (Bakhtian et al., 2017; Galler et al., 2018). In particular, tissue damage in periodontal lesions develops via an inflammatory response of the host to the microbes and to their metabolic byproducts (Bajic et al., 2015). Therefore, the essential role of oral microbiota has been established in pregnancy-associated dysbiosis.

The variations in microbiota during pregnancy have been investigated using various techniques, from cultivation to next-generation sequencing (Nuriel-Ohayon et al., 2016). Early reports revealed a significant increase of *Prevotella intermedia* sensu lato during the second trimester (Kornman and Loesche, 1980), and follow-up studies reported that enrichment of some bacterial species such as *Porphyromonas gingivalis* is associated with increased serum levels of progesterone and estrogen, which are involved in the fumarate reductase system of the pathogen (Kornman and Loesche, 1982). Compared to non-pregnant women, levels of the pathogenic bacteria *P. gingivalis* and *Aggregatibacter actinomycetemcomitans* in gingival sulcus were significantly higher during the early and middle stages of pregnancy (Fujiwara et al., 2015). Recently, a sequencing research study demonstrated high levels of gram-negative facultative bacterial species in subgingival microbiome during pregnancy (Paropkari et al., 2016). However, not all studies have corroborated the effect of pregnancy on the preferential colonization by certain bacterial species (Kumar, 2013). Our understanding of oral ecosystem, host–microbe interplay in mediating homeostasis, and ecological shifts toward disorders remains limited.

In addition to the studies mentioned above, multiple studies have employed subgingival plaques as sample sites because of their etiological relationship with periodontal diseases. A supragingival site, as a host-friendly sampling strategy for oral microbiota, is a type of habitat that has rarely been sampled although the supragingival site and periodontal pocket have similarity in bacterial compositions (Ximénez-Fyvie et al., 2000). A good supragingival plaque control has a marked effect on the subgingival microbiota of the periodontal pocket (Govitvattana et al., 2013), indicating a correlation between the two habitats. To the best of our knowledge, the microbiota composition and structural shift in supragingival plaques throughout gestation remain poorly understood. Given that supragingival plaques are more frequently flushed with saliva, the current hypothesis is that salivary sex hormones contribute to alterations in supragingival microbiota.

Compared with traditional methodologies focused on a limited number of bacterial targets, high-throughput sequencing technologies facilitate a “microbiome-wide association study” with higher resolution, sufficient depth, and fewer biases (Gilbert et al., 2016). For the study, this kind of strategy was employed to longitudinally explore the bacterial diversity and ecological shifts in the supragingival plaques of pregnant women. The relative abundance and co-occurrence networks of bacteria were compared between pregnant and non-pregnant women. Specific microbial lineages and potential genera that may correlate with pregnancy and sex hormones were also explored. These findings may provide a broader understanding of the host-microbe interaction and the ecological events leading to pregnancy-associated dysbiosis.

## Methods

### Study population

This study was approved by the Ethics Committee of Shanghai Ninth People's Hospital affiliated with Shanghai Jiao Tong University, School of Medicine, China (Document No. 201262), and conducted in accordance with the Declaration of Helsinki. All participants provided written informed consent before enrolment.

The participants constituted two groups: 11 pregnant women who were recruited during their regular visits to the Obstetrics & Gynecology Hospital of Fudan University and seven healthy non-pregnant volunteers from Shanghai Jiao Tong University School of Medicine who participated as the control subjects. The inclusion criteria were as follows: (i) presence of 28 natural teeth excluding third molars; (ii) maintenance of good oral hygiene; (iii) gestation <42 weeks and term delivery without complications for the pregnant group or a regular menstrual cycle for the non-pregnant group; and (iv) good systemic health (i.e., no diabetes, endocrine disorders, or hypertension). The exclusion criteria included the following: (i) antibiotic or steroid hormone use within the preceding 3 months; (ii) untreated oral lesions that could be clinically diagnosed (i.e., dental caries, chronic periodontitis, periapical abscess, or severe halitosis); (iii) previous diagnosis of Sjögren's syndrome or any disease characterized by xerostomia; and (iv) smoking or drinking habits. Demographics were acquired using self-reported questionnaires. Clinical examinations and evaluations were performed at the sites of the following teeth: #3, #8, #14, #19, #24, and #30. The clinical indices included plaque index (PLI), gingival index (GI), and sulcus bleeding index (SBI).

### Microbial and salivary collection

For each of the pregnant women, supragingival plaque samples, and salivary samples were collected during four visits: the first trimester (P1: 11–14 weeks gestation), second trimester (P2: 20–25 weeks), third trimester (P3: 33–37 weeks), and postpartum period (P4: 6 weeks after childbirth). Non-pregnant volunteers also complied with the four visitation times at the same time intervals (NP: NP1–NP4) and were sampled during the luteal phase of the menstrual cycle. One pregnant woman was lost to follow-up after delivery. Thus, 71 samples were gathered at the end of the follow-up period.

Sampling was performed 2 h after eating in the morning, according to the methods described in the Manual of Procedures for the Human Microbiome Project (http://hmpdacc.org/tools_protocols/tools_protocols.php) with minor modifications. After rinsing, 1.5 mL of unstimulated saliva was aspirated with a disposable sterile syringe (with the tip removed) into a sterile Eppendorf tube. Sex hormones including salivary oestradiol and progesterone were measured using a kit from Demeditec (Kiel Wellsee, Schleswig-Holstein, Germany). The selected teeth (#3, #8, #14, #19, #24, and #30) were isolated with cotton rolls and gently air-dried. A sterile Gracey curette was used to collect a pooled supragingival plaque sample from the buccal surfaces of the selected teeth. The collected plaque samples from each participant were separately stored in a sterile Eppendorf tube containing 300 μL of thioglycollate medium, transported on ice to the microbiology laboratory, and kept frozen at −20°C until use.

### DNA extraction, amplification, and sequencing

Total bacterial genomic DNA was extracted using the QIAamp DNA Mini Kit (Qiagen, Valencia, CA, USA). The DNA quality was evaluated by measuring the absorbances at 260/280 and 260/230 nm by spectrophotometry (NanoDrop® ND-1000; Thermo Scientific, Wilmington, DE, USA). Only DNA samples with A260/A280>1.7 and A260/A230>1.8 were used for further analysis (Wang et al., 2016).

PCR amplification of the 16S rDNA hypervariable V4–V5 region was carried out using the forward primer 515F (5′-GTG CCA GCM GCC GCG GTA A-3′) and reverse primer 926R (5′-CCG TCA ATT YYT TTR AGT TT-3′). A sample-unique 8-base barcode was incorporated into each forward primer to identify the sequences from the different samples. The PCR programme began with an initial denaturation for 3 min at 98°C, followed by 25 cycles of denaturation (30 s at 98°C), annealing (30 s at 50°C), and extension (30 s at 72°C) with a final extension for 5 min at 75°C. The PCR products were separated by 2% agarose gel electrophoresis, and after purifying with Agencourt AMPure Beads (Beckman Coulter, Indianapolis, IN), the final DNA concentrations were determined with the Pico-Green kit (Invitrogen, Carlsbad, CA, USA). Amplicons from different samples were mixed at equimolar concentrations for sequencing, which was performed on an Illumina MiSeq PE300 sequencing platform (San Diego, CA, USA).

### Data processing and bioinformatics analysis

Raw sequencing data were processed with the pipeline tools QIIME (version 1.7.0, http://qiime.org/) and MOTHUR (version 1.31.2, http://www.mothur.org/). To retain high-quality sequences, some sequences were eliminated; the eliminated sequences were <150 bp, contained ambiguous bases or homopolymeric stretches, had average Phred scores of <25, or were confirmed as chimeric artifacts (Bokulich et al., 2013). After trimming, the qualified sequences were clustered into operational taxonomical units (OTUs) using the Uclust programme of QIIME at 97% sequence similarity (Edgar et al., 2011) and were aligned against the Greengenes database (Release 13.8, http://greengenes.lbl.gov). After removing the singleton OTUs, a modified OTU table was generated for downstream analysis.

The rarefaction curves were constructed at 97% inter-sequence similarity using QIIME. Venn diagram and alpha diversity indexes were analyzed using MOTHUR, and the significant difference was assessed with the Dunn's multiple comparisons test using the GraphPad Prism software (La Jolla, CA, USA). To perform the beta diversity analysis, one representative sequence per OTU was selected to generate a phylogenetic tree using ARB software (Ludwig et al., 2004). The resulting tree, together with the sequence abundance data, were used for a UniFrac analysis based on a weighted matrix of distance in QIIME: the principal coordinates analysis (PCoA) (Lozupone et al., 2007). A linear discriminant analysis (LDA) effect size (LEfSe, http://huttenhower.sph.harvard.edu/galaxy) method was used to find biologically relevant features, which underlines biological consistency, statistical significance, and effect relevance (Segata et al., 2011). The Kruskal–Wallis rank-sum test was used to detect taxa with significantly different abundances with an α value < 0.05. To estimate the effect size and ranking of each taxon, LDA was applied. The threshold of the logarithmic LDA score for discriminative features was >2.0. A high LDA score indicated a significantly higher abundance of certain taxa (Guerrero-Preston et al., 2016). A partial least squares discriminant analysis (PLS-DA) was used to discriminate groups by pregnancy (Pérez-Enciso and Tenenhaus, 2003). It was performed via the mixOmics package (version 6.0.0) using R software (version 3.2.0). Variable importance in projection (VIP) >1 was used to select key variables, which were the most important contributors to generation of the model (Chen et al., 2011). Co-occurrence networks of the 50 most abundant genera were analyzed using Mothur and visualized via Cytoscape (http://www.cytoscape.org/). Spearman's correlation coefficients were also calculated. Edges were set among the genera for which the ρ-value was >0.6 and significant (*P* < 0.01). Redundancy analysis (RDA) was performed using CANOCO for Windows 4.5 (Microcomputer Power, NY, USA).

## Results

### Subject characteristics and sequence datasets

The subjects were Chinese and lived in Shanghai for many years. The demographic and clinical characteristics are listed in Table 1. The pregnant women had higher scores for PLI, GI, and SBI, indicating that the pregnant women had relatively more severe gingival inflammation. However, the subjects were still periodontitis-free as the scores were within the reference range. There was a similar trend of variations in progesterone and oestradiol during pregnancy and the postpartum period (Table S1). The hormone levels of the 3rd trimester were the highest in the pregnant group, whereas there were no significant differences from the 1st visit to the 4th visit in the non-pregnant group.

**Table 1:** Demographics and clinical status of the study population.

	**Pregnant subjects (*n* = 11)**	**Non-pregnant subjects (*n* = 7)**	***P-*value**
**DEMOGRAPHICS**			
Age (year)	27.00 (24.00, 29.00)	25.00 (25.00, 26.00)	0.44
BMI (kg/m^2^)	20.03 (19.53, 20.55)	19.53 (19.20, 20.13)	0.24
**PERIODONTAL CHARACTERISTICS**			
**1st visit**			
PLI	1.00 (0.86, 1.03)	0.89 (0.81, 0.94)	0.30
GI	0.78 (0.78, 0.89)	0.67 (0.61, 0.72)	0.01
SBI	1.11 (1.03, 1.11)	0.89 (0.78, 0.94)	0.01
**2nd visit**			
PLI	1.06 (1.00, 1.11)	0.89 (0.83, 0.94)	0.03
GI	1.00 (1.00, 1.00)	0.67 (0.61, 0.75)	0.00
SBI	1.11 (1.08, 1.22)	0.89 (0.89, 1.00)	0.01
**3rd visit**			
PLI	1.00 (1.00, 1.14)	0.89 (0.86, 1.00)	0.04
GI	1.11 (1.00, 1.19)	0.67 (0.61, 0.83)	0.00
SBI	1.22 (1.11, 1.22)	0.89 (0.89, 1.00)	0.01
**4th visit**			
PLI	1.11 (1.08, 1.22)	0.89 (0.86, 1.03)	0.01
GI	1.11 (1.00, 1.22)	0.78 (0.61, 0.78)	0.00
SBI	1.11 (1.11, 1.23)	1.00 (0.89, 1.11)	0.07

*BMI, body mass index; PLI, Plaque index; GI, Gingival index; SBI, Sulcus bleeding index. Values represent the median with the interquartile range. Statistical differences were calculated with the Mann-Whitney U-test*.

Illumina MiSeq sequencing produced 3,334,433 raw sequences, and after pre-processing, 3,053,338 usable high-quality sequences with an average of 43,005 ± 15,560 sequences per sample remained in the dataset (Figure S1A). The average length of the sequence was 392 bp without the primers, ranging from 388 to 426 bp (Figure S1B). After removing the low-credibility OTUs, taxonomic assignment of the sequences resulted in the identification of a total of 554 OTUs in the supragingival microbiota. The 200 most abundant OTUs represented 98.76% of all sequences.

### Bacterial diversity and community structure of supragingival microbiota

**Figure 1:**
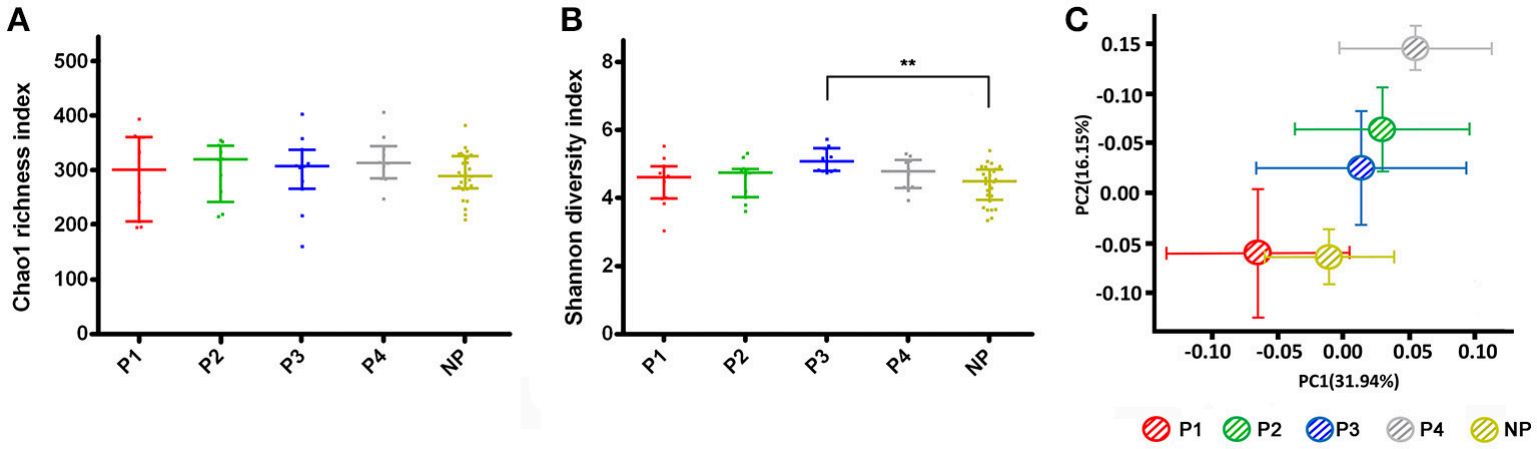
Alpha and beta diversity estimates. **(A)** Chao 1 and **(B)** Shannon index for pregnant and non-pregnant groups. The data were calculated with the Dunn's multiple comparisons test and are expressed as the median with the interquartile range. **(C)** Principal coordinates analysis (PCoA) plot. Each point represents the mean principal component score of individuals in a group, and the error bar represents the standard deviation. (“^**^” 0.001 < *P* < 0.01).

The community richness, as estimated by the Chao 1 index, was similar among the groups (Figure 1A). The Shannon index, an alpha diversity estimator, was higher in P3 than in P1, P2, P4, and NP, and there were a significant difference between P3 and NP per the Dunn's multiple comparisons test, which indicated higher bacterial diversity of the supragingival microbiota in P3 (Figure 1B). The data from the non-pregnant group were merged as a whole due to the lack of a significant difference after the calculation (*P* > 0.05). The eight rarefaction curves for P1–P4 and NP1–NP4 stabilized at >150 OTUs with 20,000 sequences, showing that the depth of sequencing was close to saturation and included most species found in the samples (Figure S2). Compared with the non-pregnant groups, P2 and P3 had a higher number of OTUs. These results were corroborated by the Venn diagram (Figure S3), which also revealed a higher number of OTUs in the pregnant group (mean = 553) than in the non-pregnant group (mean = 505). Specifically, the pregnant group had a higher number of OTUs at each given time point.

Structural similarity was explored with a PCoA of the beta diversity analysis (Figure 1C), which contained the first two principal coordinate axes (factors). Factor 1 accounted for 31.94% of the total variation, whereas factor 2 explained 16.15% of the variation. The clustering and ordination were correlated with biotic and abiotic variables that control the microbial community composition (Barberán et al., 2012). The plot of the non-pregnant group indicated similar microbial structure during four visits (Figure S4). Although the non-pregnant and P1, P2, and P3 samples partly overlapped, a clear segregation of the five bacterial communities could be seen. In addition, differences between the trimesters still existed when gestation was prolonged.

### Relative abundance and distribution of individual taxa between two groups

To characterize the bacterial distribution, the plaque microbiota of the pregnant and non-pregnant women was analyzed in terms of relative taxonomic abundance. A total of 17 phyla, 28 classes, 51 orders, 98 families, 172 genera, and 554 species were observed. Figure S5A shows the phylum structure of the oral microbiota for each participant during the four time intervals. Of the major phyla, *Proteobacteria* (27.80% of the total OTUs achieved from the pregnant group), *Bacteroidetes* (24.93%), *Firmicutes* (20.48%), *Fusobacteria* (13.48%), and *Actinobacteria* (11.95%) were the most abundant in the pregnant group, together accounting for 98.63% of the total sequences. In the non-pregnant group, the top five phyla were *Firmicutes* (29.76%), *Proteobacteria* (21.44%), *Bacteroidetes* (21.44%), *Actinobacteria* (13.65%), and *Fusobacteria* (13.13%). These phyla were present in all participants at all four sampling times, constituting the predominant supragingival phyla (Figure S5B). The top 50 genera are displayed in the heat map (Figure S5C). Of these, *Streptococcus* (11.60%) was the most abundant genus in the pregnant group, followed by *Neisseria* (11.57%), *Capnocytophaga* (9.03%), *Prevotella* (8.23%), *Fusobacterium* (7.36%), *Leptotrichia* (6.06%), *Porphyromonas* (4.27%), *Corynebacterium* (3.76%), *Veillonella* (3.22%), and *Actinomyces* (3.04%), together comprising 68.06% of the total OTUs. In the non-pregnant group, the top 10 genera were the same as in the pregnant group, and together, they comprised 72.15% of the total OTUs.

### Comparison of the microbial phylotypes responding to pregnancy

**Figure 2:**
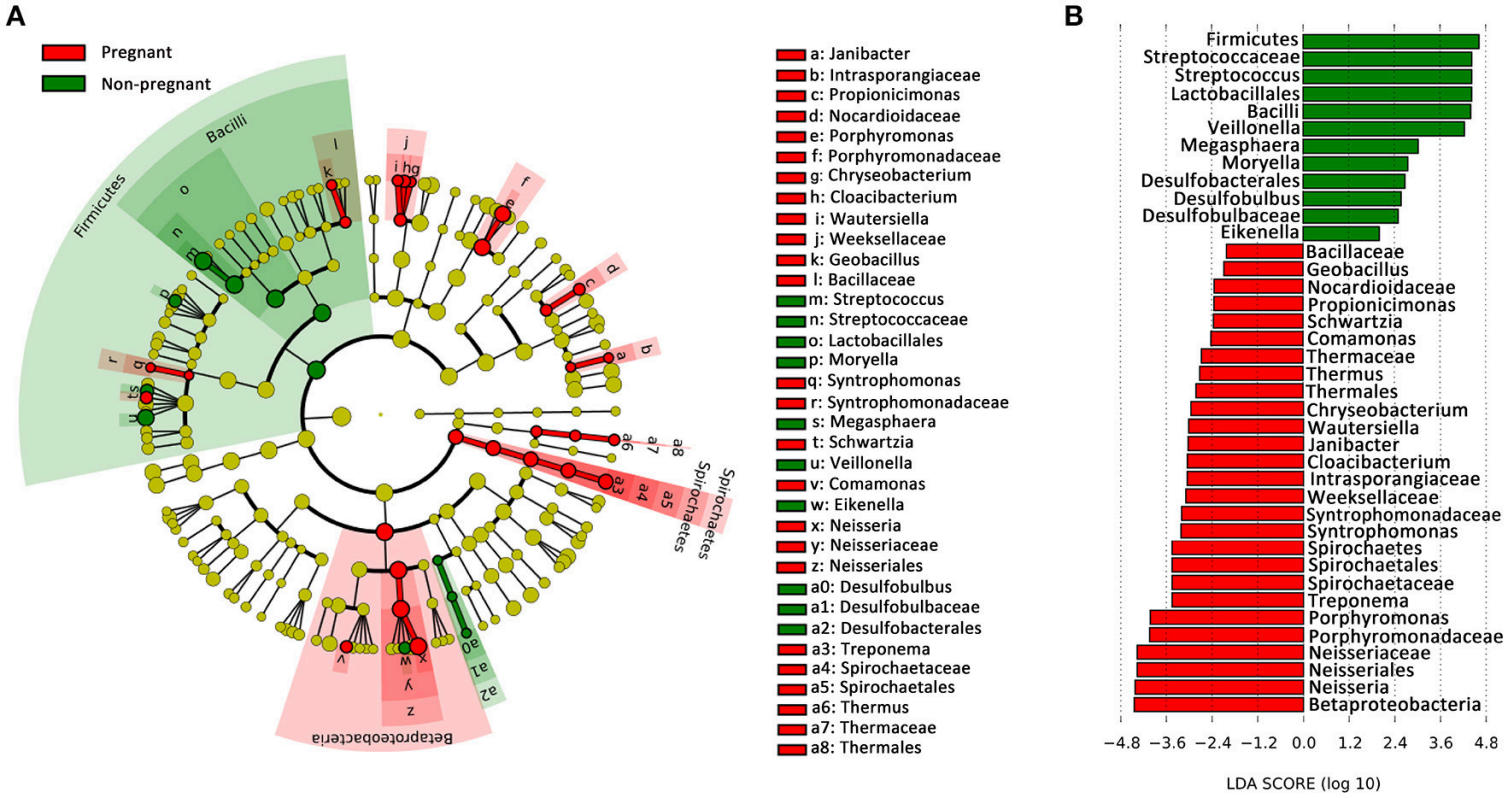
Bacterial phylotypes whose abundances were changed with pregnancy, as identified by LEfSe. **(A)** A cladogram for taxonomic representation is shown in the panel. Red indicates enrichment in samples from pregnant women, and green indicates the taxa enriched in the samples from non-pregnant women. **(B)** A histogram of the linear discriminant analysis (LDA) scores was calculated for the selected taxa.

To identify the distinguishing phylotype that most likely explained the differences between the plaque samples from the pregnant and non-pregnant groups, LEfSe was performed. A circular cladogram was generated to show the differentially abundant taxa (Figure 2A), and LDA coupled with the effect size measurement was used to assess the effect size of the taxa (Figure 2B). Of the altered families in the pregnant group, *Neisseriaceae* and *Porphyromonadaceae* and *Spirochaetaceae* were significantly enriched, and there were other six significantly different families. Furthermore, the following species were more predominant in the supragingival plaques of the pregnant women than the non-pregnant women: several aerobic genera, including *Neisseria* and *Janibacter*; facultatively anaerobic genera such as *Cloacibacterium*; and obligate anaerobic genera including *Porphyromonas, Treponema*, and *Syntrophomonas*. The microbiota of non-pregnant women was enriched with genera mainly belonging to the *Firmicutes* phylum, namely, *Streptococcus, Veillonella, Megasphaera*, and *Moryella*. Among the genera over-represented in the non-pregnant group, *Streptococcus* exhibited the highest effect size and was closely followed by *Veillonella*. The effect size and ranking of the LDA score indicated that differential enrichment of bacteria owing to different host environment (LDA >2, *P* < 0.05).

### Identification of the key genera responsible for structural differentiation

PLS-DA was applied to identify key genera that were responsible for structural differentiation due to host variations. Parameter VIP was used to select variables with the most significant contribution to discriminating the community structure. Thirty-three genera with VIP >1 were identified as key genera for separating the supragingival microbiota between the pregnant and non-pregnant groups (Table 2). Twenty-five genera were associated with the bacterial community of pregnancy, all of which were distributed across the following phyla: *Bacteroidetes* (5 genera), *Proteobacteria* (9), *Actinobacteria* (5), *Fusobacteria* (1), *Spirochaetes* (1), and *Firmicutes* (4). The table also shows eight genera that were enriched in the healthy non-pregnant group: *Moryella, Megasphaera, Veillonella, Parvimonas, Eikenella, Haemophilus, Bacteroides*, and *SHD-231*; these genera were identified as health-related bacteria (Wu et al., 2013). In particular, four genera ranked as the top two genera in the non-pregnant group (*Moryella* and *Megasphaera*) and pregnant group (*Porphyromonas* and *Neisseria*).

**Table 2:** Key genera responsible for differential distributions based on the PLS-DA.

**Genus**	**VIP score**	**Group**
*Moryella*	2.25	NP
*Megasphaera*	2.05	NP
*Porphyromonas*	2.05	P
*Neisseria*	1.96	P
*Capnocytophaga*	1.90	P
*Aggregatibacter*	1.80	P
*Propionivibrio*	1.72	P
*Lautropia*	1.72	P
*Veillonella*	1.67	NP
*Janibacter*	1.58	P
*Parvimonas*	1.56	NP
*Leptotrichia*	1.52	P
*Propionicimonas*	1.50	P
*Pseudomonas*	1.49	P
*Treponema*	1.40	P
*Lactobacillus*	1.37	P
*Bacillus*	1.35	P
*Propionibacterium*	1.29	P
*Cardiobacterium*	1.26	P
*Filifactor*	1.24	P
*Odoribacter*	1.23	P
*Eikenella*	1.23	NP
*Schwartzia*	1.20	P
*Paludibacter*	1.16	P
*Haemophilus*	1.13	NP
*Chryseobacterium*	1.12	P
*Bacteroides*	1.10	NP
*Atopobium*	1.09	P
*Kocuria*	1.08	P
*Acinetobacter*	1.07	P
*SHD-231*	1.07	NP
*Bergeriella*	1.03	P
*Comamonas*	1.03	P

*VIP, Variable importance in projection; P, Pregnant group; NP, Non-pregnant group*.

### Co-occurrence network of the most abundant genera in the supragingival microbiota

**Figure 3:**
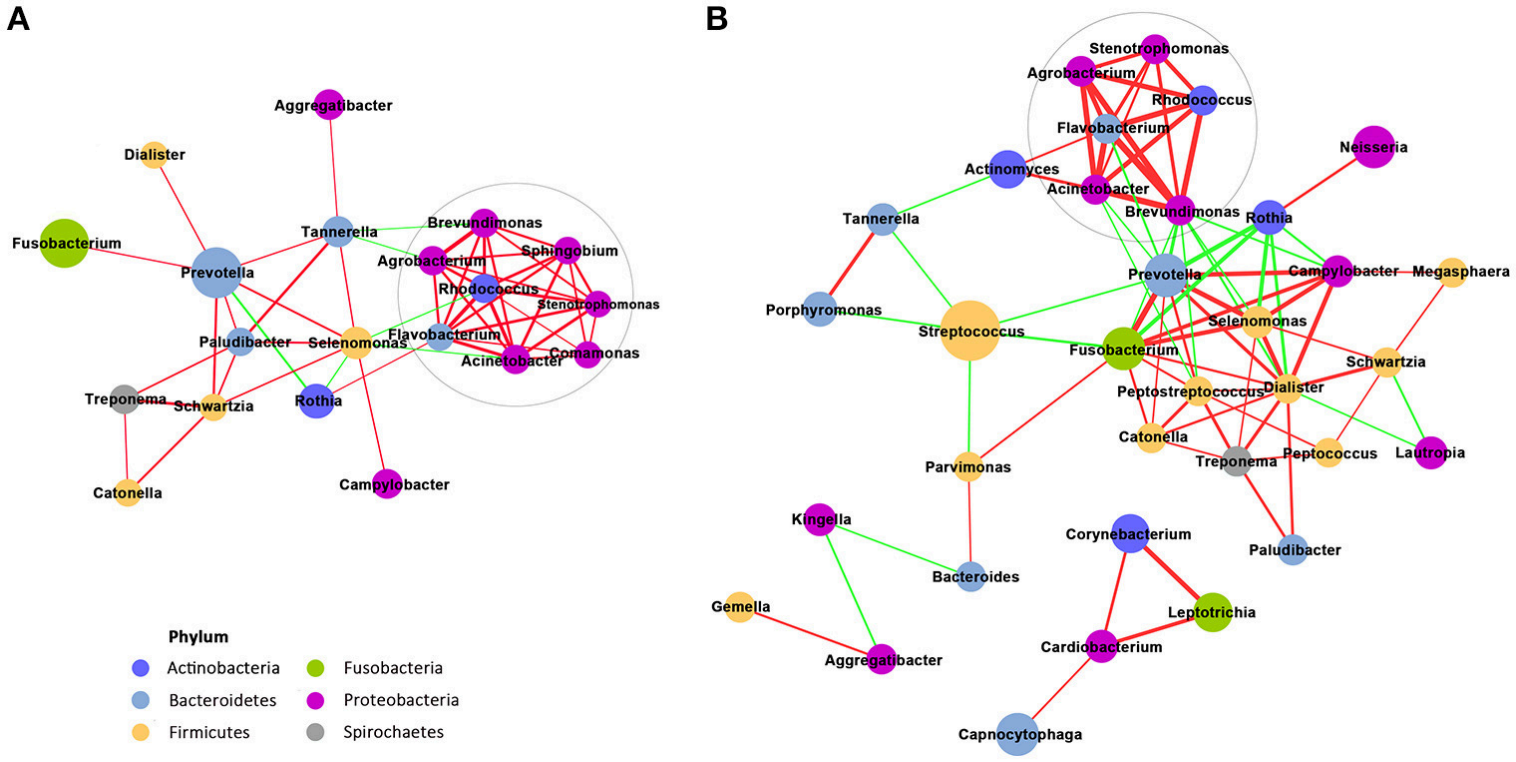
Co-occurrence network of the genera that are highly abundant in supragingival microbiota. The pregnant group is shown in **(A)**, and the non-pregnant group is shown in **(B)**. The size of the node indicates the mean relative abundance of the corresponding genus. The same color represents the genera belonging to the same phyla. The thickness of a connecting line corresponds to the coefficient values (p > 0.6), and the color of the line, namely, red or green, indicates a positive or negative correlation, respectively.

The top 50 genera in the relative abundance ranking were selected from the two groups. Genera that met the threshold of Spearman's ρ > 0.6 and *P* < 0.01 are shown in the networks (Figures 3A,B). Compared with the non-pregnant group, the positive and negative relationships of the genera in the pregnant group were more simplified. One main cluster (>5 nodes and connected with intense lines) was evident in the samples from the pregnant women, while two main clusters were evident in the samples from the non-pregnant women. From a macroscopic perspective, it was noteworthy that the genera in the clusters indicated in gray circles all exhibited a positive relationship among the genera and mainly belonged to the *Proteobacteria* phylum. *Prevotella* was the most dominant genus in the co-occurrence network of the pregnant group. Its negative relationship with the other genera was decreased, and its positive relationship was strengthened, compared with the non-pregnant group. As the most abundant genera in the non-pregnant group, *Streptococcus* showed mutual restriction with *Fusobacterium, Porphyromonas, Tannerella, Prevotella*, and *Parvimonas*.

### Correlation between supragingival microbiota and hormonal fluctuations

**Figure 4:**
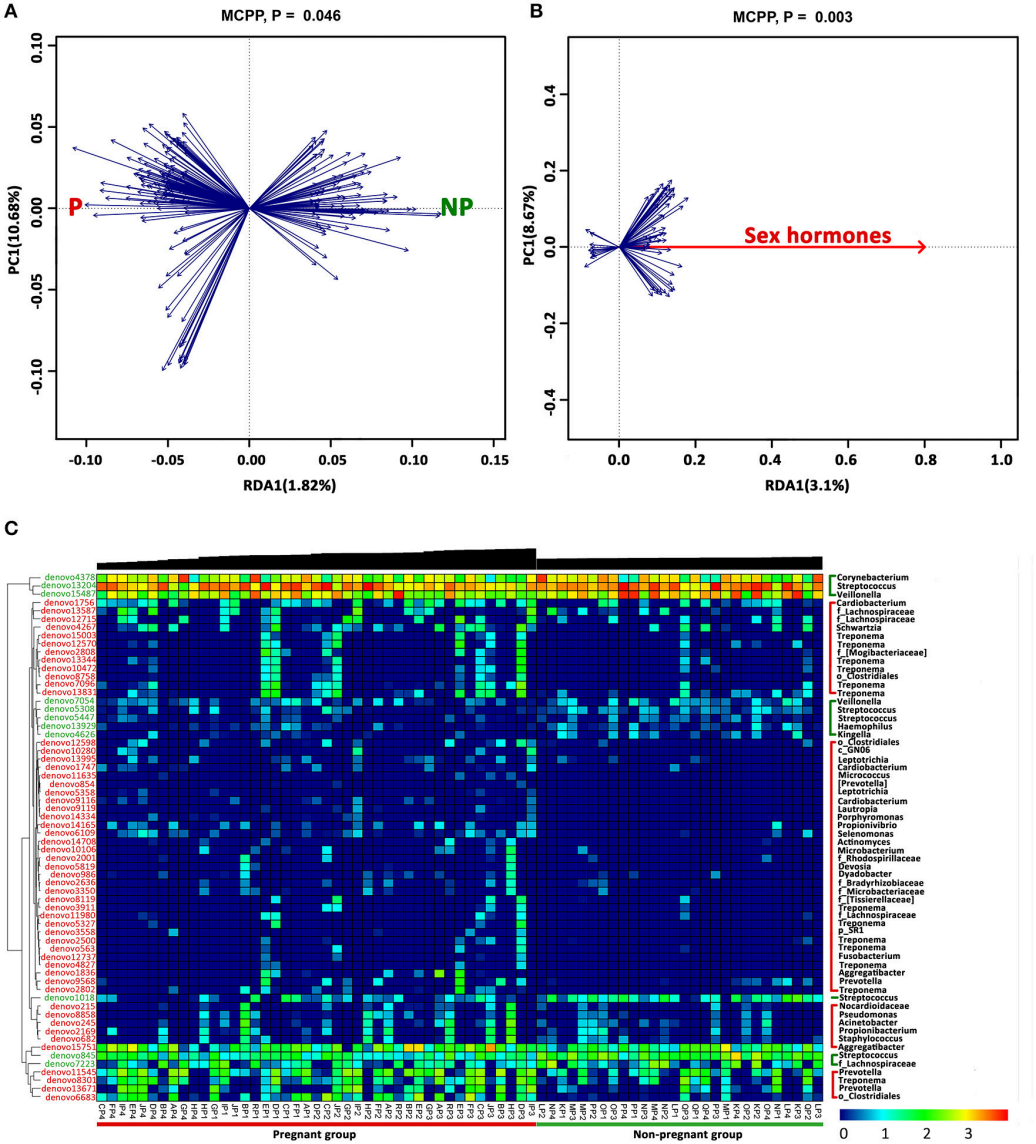
OTUs associated with sex hormones identified by two-step redundancy analysis (RDA). **(A)** The first biplot of the RDA is constrained by pregnancy as its explanatory variables. Responding OTUs that have 1.82% of the constrained variance of the supragingival microbiota are indicated by blue arrows. **(B)** The second biplot of the RDA shows the relationship between pregnancy-associated OTUs identified in **(A)** and sex hormones. Responding OTUs that have 3.1% of the constrained variance are indicated by blue arrows. **(C)** The heat map of the 64 key OTUs identified in the two-step RDA. The phylogenetic tree of the OTUs on the left is constructed in ARB based on distribution similarity. The OTUs shown in red indicate that they have higher relative abundances in the pregnant group than in the non-pregnant group, and the green OTUs are more abundant in non-pregnant group than in the pregnant group. Individuals at the bottom are arranged according to different trimesters, and accordingly, the black bars above indicate the log-transformed sex hormones levels. The color intensity of the spot represents the relative ratio of the OTUs in each sample. The taxonomic names of the OTUs are listed on the right.

A two-step redundancy analysis (RDA) was performed to explore whether a correlation between specific genera and hormonal fluctuations existed. At the first step of the RDA, non-pregnant or pregnant women were used as the constrained explanatory variables, and the relative abundances of all OTUs were used as the response variables (Figure 4A). The Monte Carlo Permutation Procedure (MCPP) showed that the constrained ordination model by the non-pregnant or pregnant group was significant (*P* = 0.046). One hundred ninety-five OTUs were identified as key variables associated with pregnancy, which explained 1.82% of the constrained variance. In the second step of the RDA, sex hormones were used as the constrained explanatory variables, and the relative abundances of the OTUs identified in the first step of the RDA were used as the response variables (Figure 4B), thus generating 64 OTUs that were closely relevant to the sex hormones. Blue arrows pointing in the same direction as the sex hormones were predicted to have a positive correlation. The MCPP showed that the model was also significant (*P* = 0.003). The heat map displays the dynamic variation in the selected OTUs and sex hormones (Figure 4C). Fifty-three OTUs, which mainly belonged to genera *Treponema* (12 OTUs), *Prevotella* (4 OTUs), *Cardiobacterium* (3 OTUs), *Aggregatibacter* (2 OTUs), *Leptotrichia* (2 OTUs), other bacteria of the family *Lachnospiraceae* (3 OTUs), or other bacteria of the order *Clostridiales* (3 OTUs), were more abundant in the plaques of the pregnant women. In contrast, 11 OTUs were observed to have negative correlations with sex hormones, among which 5 OTUs belonged to *Streptococcus* and 2 OTUs belonged to *Veillonella*.

## Discussion

Recent advances in microbial-surveying techniques and bioinformatics have made sensitive detection, high-resolution discrimination and deep characterization of bacterial communities possible (Hamady and Knight, 2009). This study, which was carried out using Illumina MiSeq sequencing to characterize the supragingival microbiota of pregnant women in comparison with non-pregnant individuals, showed clear differences in the community structure and composition that were previously not evident using experimental approaches. As a longitudinal study, this work confirms previous findings that there is a group of core species (*Proteobacteria, Firmicutes, Bacteroidetes, Fusobacteria*, and *Actinobacteria*) in a healthy oral cavity (Li et al., 2013; Utter et al., 2016), and provides a further understanding of host-induced alterations in the bacterial community by factors such as female sex steroids during pregnancy.

The starting population differences were minimized as much as possible according to the inclusion and exclusion criteria. A community-wide range of microbiota was explored in this study. Based on the description in the alpha-diversity index (e.g., richness and diversity estimators and rarefaction curves), it can be concluded that P3 has the highest diversity. Basing the calculation on singleton and doubleton species (Chao, 1984), the Chao 1 richness estimator showed no difference in the number of rare species between the pregnant and non-pregnant groups. The tendencies of the hormonal surges of progesterone and oestradiol were consistent, showing a 10-fold increase in the 3rd trimester compared with the 1st trimester in the saliva of the pregnant group. Therefore, it is hypothesized that pregnancy creates a nutrient environment that is more favorable to some sensitive strains. In addition, as previously reported, PCoA reveals the effects of more transient factors related to nutrient availability (Lozupone et al., 2007). The non-pregnant group tended to cluster away from the pregnant group with a small standard deviation, suggesting that the gestation period has an impact on the microbial community and acts as a driver of compositional differentiation.

A majority of the observed taxa were present in both the pregnant and non-pregnant groups, albeit at different frequencies of detection. The taxa of the samples were distributed in 17 bacterial phyla, which included five of the most predominant phyla frequently detected in the oral cavity: *Firmicutes, Proteobacteria, Bacteroidetes, Actinobacteria*, and *Fusobacteria*; this observation was in accordance with previous studies (Dewhirst et al., 2010; Griffen et al., 2011). Zaura et al. concluded that in supragingival plaques, *Streptococcus, Capnocytophaga, Corynebacterium*, and *Fusobacterium* belonged to the major core genera (Zaura et al., 2014). The LEfSe revealed that pregnancy was associated with specific changes in the microbiota and clearly demonstrated that some taxa showed enrichment in the supragingival plaque of pregnant women. Within the phylum *Firmicutes*, the bacilli were health-related, which was consistent with the study by Griffen et al. (2012). *Streptococcus* and *Veillonella* had the highest LDA score in the non-pregnant group. In the study by Kumar et al. *Streptococcus* and *Veillonella* also accounted for a significantly greater fraction of the plaques in the healthy individuals (Kumar et al., 2005). *Streptococcus*, one of the earliest and most abundant colonizers of biofilms, can produce an antimicrobial effect against several exogenous pathogens (Watanabe et al., 2009), and *Veillonella* can utilize the lactate produced by the *Streptococcus* as an energy source (Kuramitsu et al., 2007).

In the pregnant group, *Neisseria* and *Porphyromonas* had the highest LDA score. In the subgingival microbiota of pregnant women in a previous study, *Neisseria* was significantly decreased (Paropkari et al., 2016), while in this study, it was increased. Sampling habitats may explain the discrepancies because the supragingival niche favors the growth of aerobic or facultative anaerobic species (Thurnheer et al., 2016). On the other hand, the usage of thioglycollate medium for sample storage might cause the overrepresentation of *Neisseria* in the pregnant cohort. Besides, *Neisseria* were shown to be related to periodontal inflammation (Vieira Colombo et al., 2016). *P. gingivalis* is one of the best-understood periodontal pathogens. It has been shown to trigger the variations in the abundance and diversity of the microbial structure that result in dysbiosis and unresolved inflammation (Hajishengallis et al., 2011). *P. gingivalis* can form a pathogenic consortium called the red complex together with two other species, *Treponema denticola* and *Tannerella forsythia* (Hajishengallis and Lamont, 2012). Oral *Treponema* strains can be easily isolated from subgingival plaques in patients with periodontitis (Sakamoto et al., 1999). In a study by Abusleme et al. *Treponema* was over-represented in periodontitis with the highest LDA score, and the authors inferred that the dominance of *Spirochetes* may have been due to autogenic succession (Abusleme et al., 2013). In the present study, *Treponema* had the third highest LDA score in pregnant women without periodontitis. Whether the presence of *Treponema* results in increased vulnerability of pregnant women to periodontitis needs further investigation.

Thirty-three key genera were identified to be relevant to the structural changes of pregnancy and non-pregnancy. It has been shown that PLS-DAs of the sequencing data are robust and sufficiently sensitive to identify specific members contributing to the community separation (Zhang et al., 2010). *Moryella* is a Gram-positive, anaerobic genus first reported in 2007 (Carlier et al., 2007). Its role and function have not yet been fully deciphered in the oral bacterial community. Our data showed that, despite its low relative abundance, *Moryella* was present in 25/43 samples from the pregnant group, while in the non-pregnant group, the proportion was 26/28. *Megasphaera* belongs to the *Veillonellaceae* family and has been frequently identified with culture-independent techniques. Specifically, *Megasphaera* is observed to be over-represented in periodontal disease, rendering it as a suspected disease-associated bacterium (periopathogen) (Kumar et al., 2003; Dewhirst et al., 2010). However, another study reported that the novel *Megasphaera* sp. *DISK18* is incapable of generating virulence-determining genes and degrading collagen, highlighting its non-pathogenic and commensal lifestyle (Nallabelli et al., 2016). In this study, endowed with a high VIP score and enrichment in the non-pregnant group, *Megasphaera* was assumed to be a health-related genus in supragingival plaques.

Co-occurrence and correlation patterns are used for the prediction of inter-taxa correlations in the environment. Positively correlated genera may have cooperative interactions, such as habitat affinities, metabolic exchange, and symbiotic relationships (Barberán et al., 2012). For example, the coaggregation effect of *Prevotella* and *Fusobacterium* has been demonstrated using co-culture assay (Okuda et al., 2012), which may be critical for the temporary retention of microbes on dental surfaces (Hojo et al., 2009). In this study, *Prevotella* and *Fusobacterium* also exhibited a high positive correlation coefficient value in the two groups. The cluster containing *Agrobacterium, Stenotrophomonas, Flavobacterium, Acinetobacter, Rhodococcus*, and *Brevundimonas* had a strong mutually positive connection, of which the underlying biotic mechanism is still not clear. From the network of the non-pregnant women, it is speculated that, although suspicious periodontal pathogens such as *Porphyromonas, Tannerella*, and *Prevotella* were present, there was a competition by other strains so that the host could maintain periodontal health. In particular, *Prevotella* exhibited a delicate ecological balance between mutualistic and antagonistic interactions with its counterparts in the communities of the plaques from non-pregnant women, while in the pregnant group, the antagonistic interaction of *Prevotella* was decreased, and the mutualistic relationship was strengthened. According to the network shift between pregnancy and non-pregnancy, it is assumed that pregnancy can act as a host factor to filter species that possess traits that are suitable for the habitat.

As a multivariate statistical tool, the RDA is able to identify relevant bacteria that are associated with physiological conditions without a prior hypothesis (Wang et al., 2012). Fifty-three OTUs were positively correlated and increased with female hormones, while 11 OTUs were negatively correlated. One of the underlying mechanisms, for instance, refers to oestradiol and progesterone being substituted for vitamin K in bacterial anaerobic respiration, especially for black-pigmented *Bacteroides* such as *Bacteroides melaninogenicus* and *Prevotella intermedia* (Kornman and Loesche, 1982). Some species of *Treponema* like *T. denticola* are capable of steroid metabolism as a virulence factor (Clark and Soory, 2006). Other putative periodontal pathogens, including *Porphyromonas, Aggregatibacter*, and *Fusobacterium*, were also positively correlated with reproductive hormones in present study. Paropkari et al. investigated the subgingival microbiomes of pregnant women and found that pregnancy appeared to promote colonization by species capable of estrogen metabolism (Paropkari et al., 2016). Recently, *Fusobacterium nucleatum* has received increasing attention because of its relation to adverse pregnancy outcomes (Han et al., 2010). It is still noteworthy that some species of bacteria which were not linked to hormones may have latent influences on the gingiva.

In this study, pregnant women had greater gingival inflammation, which was consistent with previous studies (Gürsoy et al., 2013). The gingival index showed that gingival inflammation in P1 was statistically more severe than NP1. However, the plaque index of pregnant women in P1 was not significantly different from that of the non-pregnant group, indicating similar oral hygiene conditions at least at 1st visit. So it was speculated that the gingiva was aggravated during pregnancy though it interacted with a normal microbial load. Based on the data above, it can be assumed that the hormonal fluctuations that occur during pregnancy have a potential effect on the composition of supragingival microbiota. Subsequently, the microbe-induced immune-inflammatory response might indirectly contribute to gingival inflammation (Wu et al., 2015).

In the current study, it is acknowledged that the sample size may have had an impact on the findings. Since this was an exploratory analysis, intriguing, and preliminary data were provided to evaluate the supragingival microbiota and its relationship with healthy pregnant women. However, it is essential to determine how pregnancy and sex hormones influence related microbes using experimental procedures. In conclusion, the present study has characterized the dynamic microbiota of supragingival plaques and provided a better understanding of the ecological and microbial shifts associated with pregnancy. This work suggests that pregnancy has a potential role in shaping supragingival microbiota and that the changes in some key populations may transform the microorganisms into an at-risk-for-harm entity, which may be related to individual susceptibility to pregnancy-associated oral dysbiosis and disease.

## Author contributions

XZ, CX, and ZH: conceived and designed the experiments. WJ, WL, and LG: performed the experiments. WL, DA, HP, and XH: analyzed the data. CN and KY: contributed reagents, materials, analysis tools. WL, CX, and ZH: Wrote the paper: WL, WJ, XH, LG, DA, HP, CN, KY, XZ, CX, and ZH: revised the manuscript.

### Conflict of interest statement

The authors declare that the research was conducted in the absence of any commercial or financial relationships that could be construed as a potential conflict of interest.
