# Supplementary material for: Ecological Shifts of Supragingival Microbiota in Association with Pregnancy
Source: Front Cell Infect Microbiol. 2018 Feb 15;8:24. doi: 10.3389/fcimb.2018.00024 (PMC5819318; doi:10.3389/fcimb.2018.00024)

Ecological Shifts of Supragingival Microbiota in Association with Pregnancy

Wenzhen Lin[1†], Wenxin Jiang[1†], Xuchen Hu[1], Li Gao[1], Dongmei Ai[2], Hongfei Pan[2], Chenguang Niu[1], Keyong Yuan[1], Xuedong Zhou[3], Changen Xu[4]*, Zhengwei Huang[1]*.

Supplementary Information

The supplementary information includes:

Supplementary Table S1

Supplementary Figure S1

Supplementary Figure S2

Supplementary Figure S3

Supplementary Figure S4

Supplementary Figure S5

Supplementary Table S1 | The concentrations of salivary hormones (pg/ml) (mean ± standard deviation).

	**1st visit**	**2nd visit**	**3rd visit**	**4th visit**
**Pregnant**				
Progesterone	243.42 ±61.39	629.29 ±81.79	2356.96 ±724.89	33.91 ±19.43
Estrogen	15.91 ±7.98	35.49 ±14.36	177.12 ±45.13	3.49 ±1.52
**Non-pregnant**				
Progesterone	94.82 ±15.22	95.64 ±21.31	104.74 ±27.21	99.41 ±18.50
Estrogen	3.58 ±0.96	3.3 ±1.41	3.74 ±1.58	4.11 ±1.92

Supplementary Figure S1 | (**a**) The number of sequences for each sample. The bars represent 18 individuals at the different phases. (**b**) Length distribution of the qualified sequences.

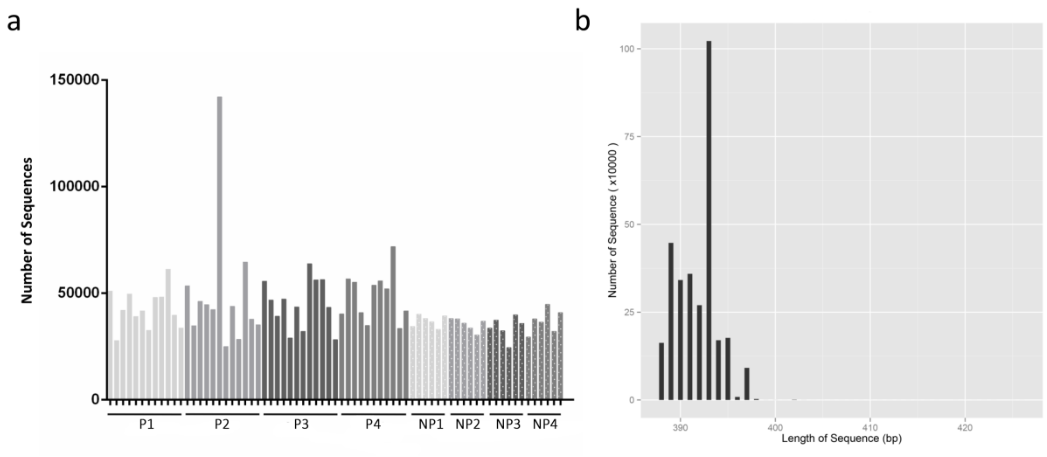

Supplementary Figure S2 | OTU rarefaction curves. Each line represents the pregnant or non-pregnant group at different time intervals.

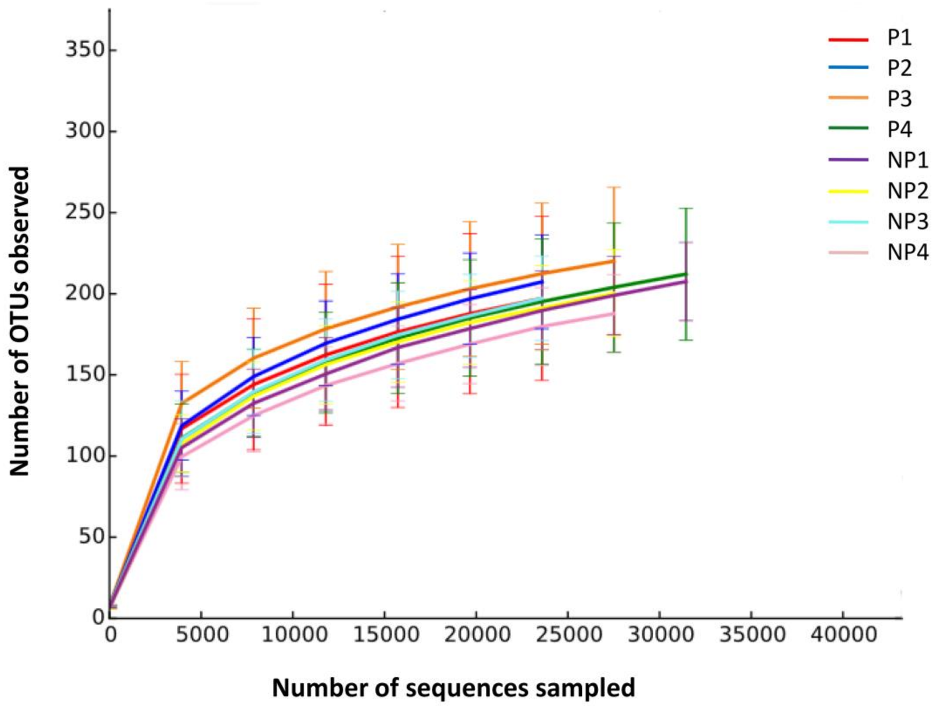

Supplementary Figure S3 | Venn diagram of the OTUs among the groups.

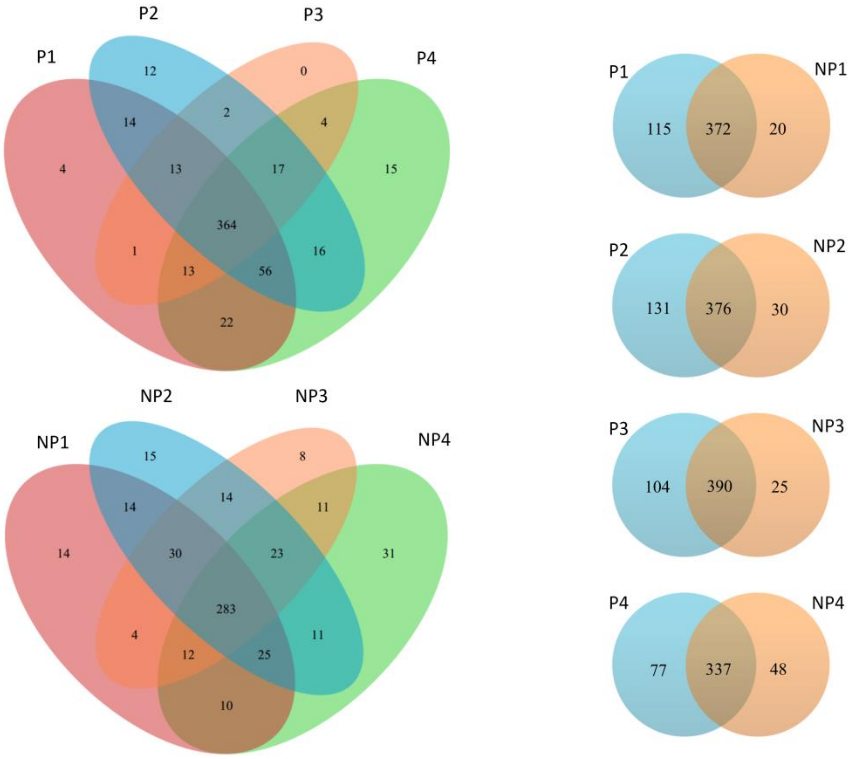

Supplementary Figure S4 | Principal coordinates analysis (PCoA) plot of the non-pregnant group.

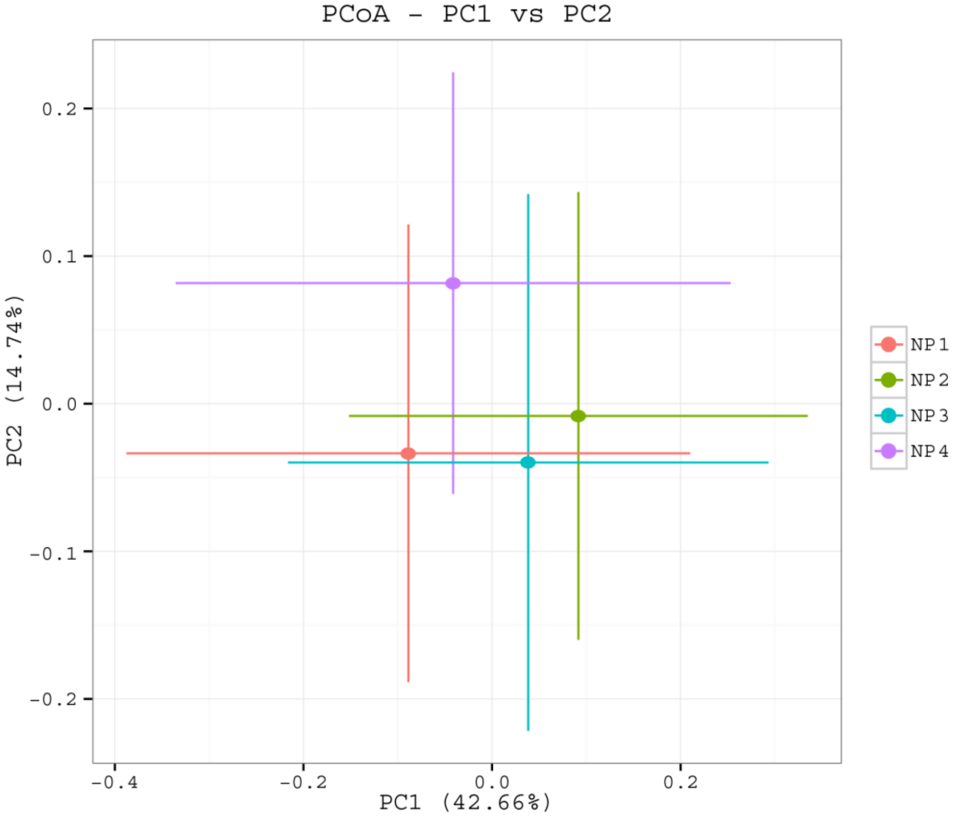

Supplementary Figure S5 | Dynamic composition shifts in the supragingival plaques. (**a**) Relative abundance of the bacterial phyla of the two groups. (**b**) The phyla core represents the shared phyla among the four time intervals for all samples. (**c**) Heat map analysis of the top 50 genera in richness.

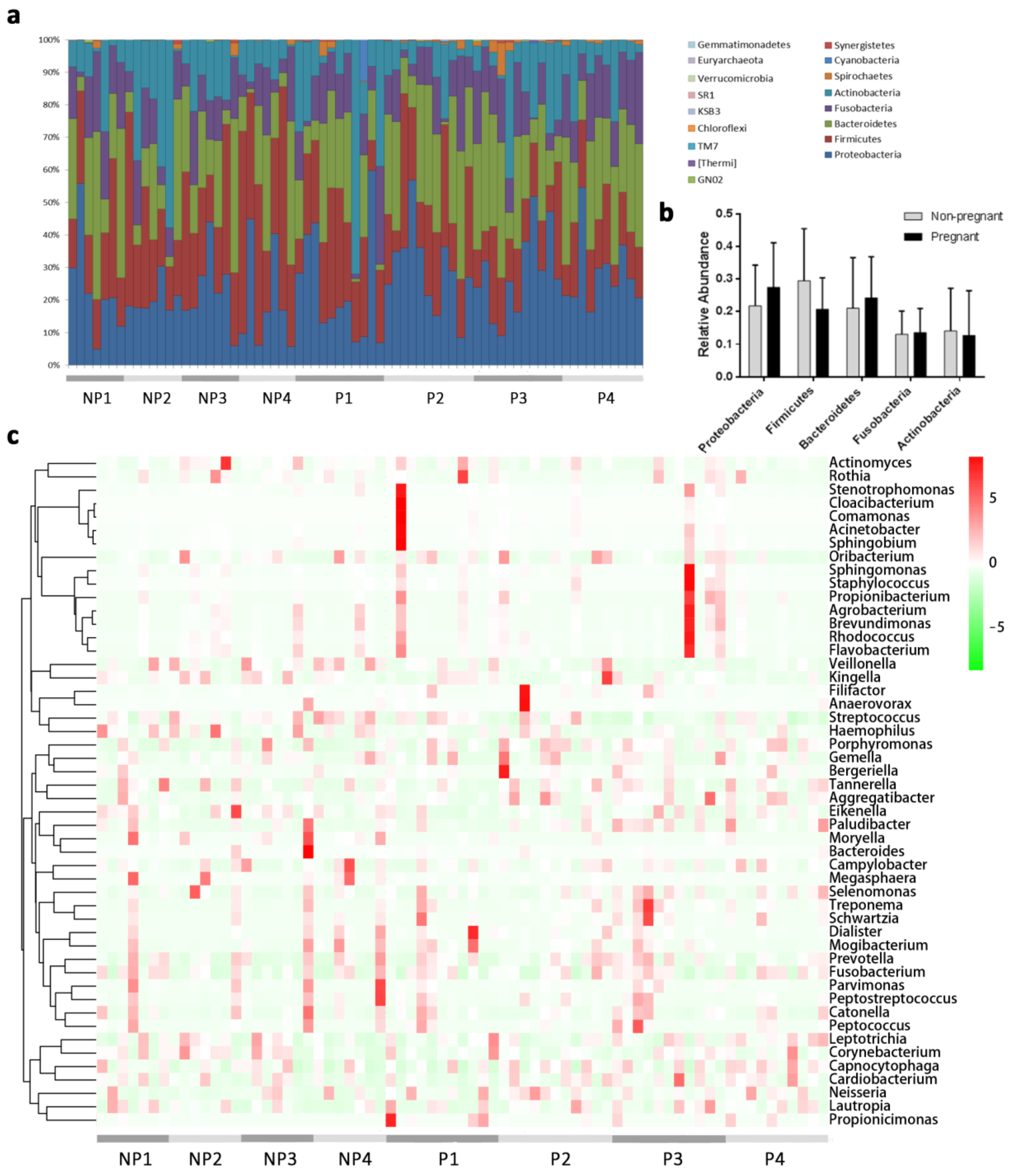

Supplement: Supplementary file 1 [file Presentation1.PDF]
